# Winter chilling regulates seedling development in Mediterranean terrestrial orchids cultivated *in vitro*

**DOI:** 10.1093/aobpla/plag039

**Published:** 2026-08-27

**Authors:** Gwenaëlle Deconninck, Argyrios Gerakis, Victoria Chatzopoulou, Alexandra Thoma, Eirini Katsalirou

**Affiliations:** Department of Food Science and Technology, Ionian University, Vergoti Street, 28100 Argostoli, Greece; Department of Biology, Lund University, Kontaktvägen 10, 22100 Lund, Sweden; Department of Food Science and Technology, Ionian University, Vergoti Street, 28100 Argostoli, Greece; Department of Food Science and Technology, Ionian University, Vergoti Street, 28100 Argostoli, Greece; Università degli Studi della Basilicata, Via Nazario Sauro 85, 85100 Potenza, Italy; Department of Food Science and Technology, Ionian University, Vergoti Street, 28100 Argostoli, Greece

**Keywords:** chilling requirement, *ex situ* propagation, Mediterranean orchids, Orchidaceae, protocorm, restoration, seedling development, temperature threshold, terrestrial orchids, winter chilling

## Abstract

Effective *ex situ* propagation is critical for the conservation and restoration of threatened terrestrial orchids. However, large-scale propagation remains constrained by developmental bottlenecks including the transition from protocorms to plantlets with storage organs. Although cold exposure is often recommended to improve propagation success, the thermal conditions governing survival and post-dormancy performance remain poorly understood. We experimentally exposed protocorms of the Mediterranean terrestrial orchids *Anacamptis coriophora*, *A. laxiflora*, *Himantoglossum hircinum*, and *H. robertianum* to eight thermal treatments for 8 weeks, including controlled laboratory conditions and outdoor sites along an altitudinal gradient. Survival, shoot growth, and tuber development were quantified before and after summer dormancy, and their relationship with temperature descriptors was analysed using a model selection approach. Plant performance was consistently better explained by cumulative chilling exposure than by mean temperature alone, with different developmental processes responding to distinct thermal thresholds. Exposure to moderately low temperatures (10°C–12°C) determined survival and reserve accumulation before dormancy, while chilling below 6°C–7°C doubled post-dormancy shoot length in responsive species, revealing carry-over effects across growing seasons. Species differed markedly in their thermal responses; increased chilling reduced post-dormancy survival by ∼50% in *A. coriophora* but increased it two-fold in *A. laxiflora*, whereas *H. robertianum* developed leaves only following cold exposure. Winter chilling therefore appears to be a key regulator of seedling development in Mediterranean terrestrial orchids. The identified thermal thresholds provide a basis for improving *ex situ* propagation and restoration protocols, while highlighting species-specific vulnerabilities and responses to future climate change.

## Teaser

Mediterranean terrestrial orchids differ markedly in how their seedlings respond to temperature during *in vitro* cultivation. Cumulative chilling predicted seedling performance better than mean temperature, with distinct temperature thresholds controlling survival, tuber development, and post-dormancy growth. The effects of chilling extended into the following growing season, highlighting the importance of considering species-specific thermal requirements to improve propagation success and inform conservation under milder winters.

## Introduction

Orchidaceae are among the most diverse and widespread angiosperm families (Swarts and Dixon 2009, Chase et al. 2015, Zhang et al. 2018). However, many orchid species are increasingly threatened due to habitat degradation, collection for commercial trade, and climate change (Vogt-Schilb et al. 2015, Gerakis et al. 2016, Hirth 2016, Kreziou et al. 2016, Popova et al. 2016, Fay 2018, Hinsley et al. 2018, Hutchings et al. 2018, Masters et al. 2020). Conservation and restoration efforts are urgently needed (Ponert et al. 2012). *In vitro* propagation represents a promising approach (Katsalirou et al. 2017, Phillips et al. 2020, Jolman et al. 2022), provided that standardized, scalable protocols are developed for adoption by plant propagation laboratories to enable mass seedling production for conservation and restoration initiatives (Stewart and Kane 2006, Lee and Yeung 2018, Deconninck and Gerakis 2021, Jolman et al. 2022).

In natural environments, temperate terrestrial orchids experience seasonal temperature fluctuations that regulate growth and developmental transitions (Arditti 1992). Replicating these cues during *in vitro* propagation is therefore essential to ensure completion of the plant life cycle. Cold stratification, defined as the exposure of mature seeds to low temperature, breaks orchid seed dormancy and promotes germination (Rasmussen 1995, Kauth et al. 2008a, Jevšnik and Luthar 2015). Germination typically occurs at ambient temperatures of ∼24°C, although optimal temperatures may vary among species according to their distribution ranges (Rasmussen 1995, Rännbäck 2007, Kauth et al. 2008a, Godo et al. 2010, Zhang et al. 2018). In some cases, fluctuating rather than constant temperatures have been shown to enhance germination in European orchids (Calevo and Bazzicalupo 2020, Nabieva et al. 2025). Prolonged cold exposure can also initiate or accelerate flowering (vernalization; Chouard 1960, Michaels and Amasino 2000, Kim et al. 2009). However, while germination and flowering induction are relatively well understood, temperature requirements during intermediate stages, particularly seedling growth and development, remain poorly characterized (Stewart and Kane 2006).

A critical stage during *in vitro* propagation of terrestrial orchids is the transition from protocorm to plantlet, during which the shoot apical meristem (SAM) is established (Yeung 2017). The protocorm describes the life stage from germination until the formation of a shoot tip with primordial leaves but no roots, during which mycorrhizal fungal association is established (Rasmussen 1995). Cold exposure treatment has been recommended to induce the transition from protocorm to plantlet in temperate orchids, including representatives of *Anacamptis* Rich., *Dactylorhiza* Neck. ex Nevski, *Himantoglossum* Spreng., *Neotinea* Rchb.f., *Orchis* Tourn. ex L. (including the former genus *Aceras* R.Br.), and *Spiranthes* Rich., with recommendations for temperature and duration ranging from 2°C to 12°C over 6–12 weeks (Hadley 1970, Beyrle et al. 1985, Rasmussen 1995, Ponert et al. 2012, Malmgren and Nyström 2026). Differences appear in cold treatment requirements that are likely to depend on individual species cold tolerance and population geographic origin (e.g. elevation or latitude) (Kauth et al. 2008, 2011, Rasmussen et al. 2015). Better definition of the temperature requirements of orchid seedlings is necessary in order to improve *in vitro* propagation protocols, for restoration programmes (including plant translocation) and to model species response to climate change (Garbisch et al. 1995, Kauth et al. 2011, Shefferson et al. 2017).

This study explores the influence of cold treatment on orchid seedling survival and growth, from protocorm until the end of summer dormancy. Protocorms of the terrestrial orchids *Anacamptis coriophora* (L.) R.M. Bateman, Pridgeon & M.W. Chase, *Anacamptis laxiflora* (Lam.) R.M. Bateman, Pridgeon & M.W. Chase, *Himantoglossum hircinum* Spreng., and *Himantoglossum robertianum* (Loisel.) P. Delforge grown asymbiotically *in vitro* were subjected to varying temperature regimes. We hypothesized that there would be a quantifiable, species-specific relationship between organ growth (tubers and shoots) and temperature of the cold treatment.

## Materials and methods

### Seed collection

**Figure 1:**
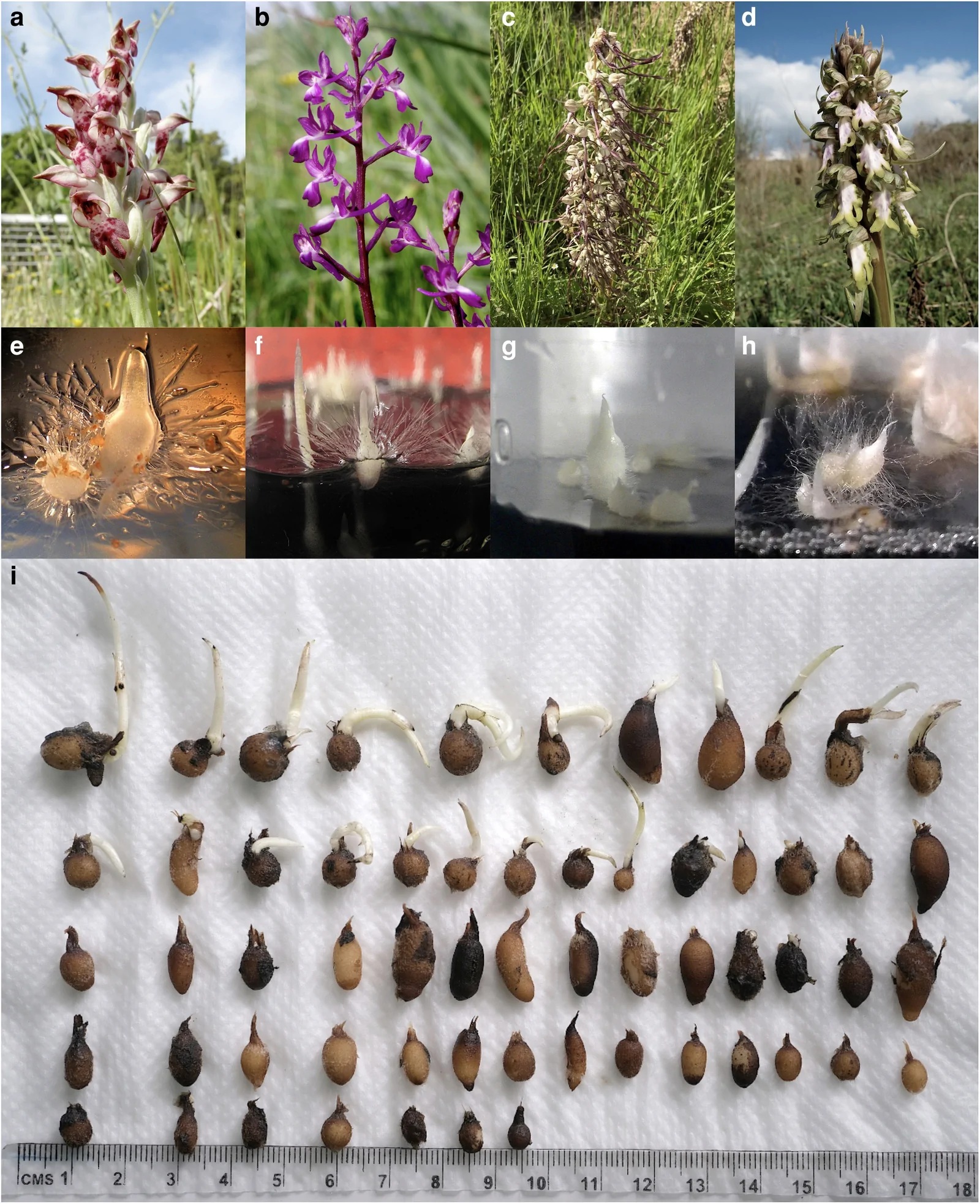
Inflorescences (top) and protocorms (middle) of *Anacamptis coriophora* (a, e), *A. laxiflora* (b, f), *Himantoglossum hircinum* (c, g), and *H. robertianum* (d, h). Bottom: Sprouting tubers of *A. laxiflora* after summer dormancy (i).

Mature seeds of *A. coriophora*, *A. laxiflora*, and *H. robertianum* were collected from healthy and robust parent plants between 2016 and 2020 on the island of Cephalonia, Greece (Table 1; Fig. 1a–d). Mature seeds of the hardier *H. hircinum* were collected in 2020 from the island of Ré, France (Table 1). The selected species, while having localized distributions, were abundant in their locations, such that seed collection would not jeopardize the natural populations. Foreign materials such as capsule fragments were removed by sieving through a 500-µm sieve. The seeds were dried in a desiccator with silica gel, sealed in labelled glass vials, and stored at −20°C until sowing.

**Table 1:** Location of seed donors, collection year, sowing date, seed disinfection duration, reflasking date, total number of protocorms, and number of experimental vessels for the four studied orchid species.

Orchid species	Location	Collection year	Sowing date	Disinfection time (min)	Reflasking date	Number of protocorms	Number of vessels
*Anacamptis coriophora*	34SDH8074312878	2017	10 Nov 2020	60	22 Jan 2021	1960	392
*Anacamptis* *laxiflora*	34SDH5698823917	2018	5 Aug 2020	85	10 Jan 2021	1600	320
*Himantoglossum robertianum*	34SDH6148321415	2016	5 Aug 2020	85	5 Jan 2021	1600	320
*Himantoglossum hircinum*	30TXS1881618746	2019	11 Mar 2020	85	31 Dec 2020	320	64

Coordinates are in the Military Grid Reference System (MGRS; https://en.wikipedia.org/wiki/Military_grid_reference_system; verified 20 January 2026).

### Laboratory method

#### Preparation of solutions

Seeds were sown asymbiotically *in vitro* in March, August, and November 2020 (Table 1). In asymbiotic propagation, instead of relying on symbiotic fungi for nutrition, the seeds are scarified using a disinfecting solution and the nutrients are furnished by a nutrient medium (Kauth et al. 2008a, Yam and Arditti 2009, Rasmussen et al. 2015; Çiǧ et al. 2018, Lee and Yeung 2018). The culture substrate was a modified version of the ‘SM-organic’ medium (Supplementary Table S1). This is a time-tested formula developed by Svante Malmgren, also published by Rasmussen (1995), which has proved successful in previous studies (Katsalirou et al. 2017, 2019, Deconninck and Gerakis 2021, Calevo et al. 2022). The term ‘organic’ refers to the N source, an amino acid mixture sold under the trade name Vaminolac® (Fresenius Kabi, Uppsala, Sweden). After mixing, pH was adjusted to 5.5–6.0 with a few drops of 3 M H_2_SO_4_ solution.

#### Sowing

Sowing was performed inside a laminar flow cabinet (Esco®, EQU/04-EBC-2A, Singapore). All surfaces and materials were disinfected (for details, see Deconninck and Gerakis 2021). For each species, 90 mg of seeds were scooped up with a spatula and placed in 15-ml Sarstedt® test tubes. The test tubes were nearly topped with 0.5% NaClO disinfecting solution and a drop of Tween® 20 (Merck, Darmstadt, Germany) to reduce surface tension, capped, and shaken vigorously to remove any air bubbles in contact with the seed. The shaking was repeated every 15 min thereafter until the end of the disinfection period. Disinfection times, specific to each orchid species (Katsalirou et al. 2017, 2019, Deconninck and Gerakis 2021), were chosen based on past experiments (Table 1). Then, the suspensions were decanted into Erlenmeyer flasks fitted with a funnel lined with filter paper to recover the seeds. The filter paper had been pre-soaked in the same disinfecting solution. After filtering the suspensions, each filter paper was rinsed five times with 7 ml of sterile deionized water. Each culture vessel (100 ml urine samplers made of polypropylene) was filled with 16 ml of sterile nutrient medium and a sterile potato cube cut from the inner tissue of an organic potato tuber (*Solanum tuberosum* L.). It is believed that potato is a natural supplement that promotes seed germination and seedling growth in orchid tissue culture (Arditti 1967, Utami and Hariyanto 2020). The seeds were scraped with a spatula from the filter paper and distributed evenly within the culture vessel, ∼4 mg of seeds per vessel. The tips of the steel tools were heat-sterilized with an alcohol burner between sowings. After sowing, the vessels were incubated in a dark room at an ambient temperature of 22°C. At regular intervals, all vessels were visually examined for the development of fungal and bacterial infections.

#### Reflasking

At the protocorm stage, the seedlings were thinned and reflasked in culture vessels (identical to those used for seed sowing) with fresh medium. Appropriately sized protocorms were defined as round protocorms that had developed a small shoot tip (Fig. 1e–h). The vessels containing the protocorms were externally disinfected with 70% v/v ethanol solution before being exposed to a UVC germicidal lamp (Philips TUV 36W/G36 T8). Vessels for reflasking were filled with 30 ml of sterile nutrient medium and a potato cube. After solidification, the protocorms were transferred with forceps from the old to the new medium. Between transfers, the tips of the forceps were wiped clean and heat-sterilized using an alcohol burner. Each new vessel hosted five randomly selected protocorms. The number of vessels for the experiment depended on the number of protocorms available for each species: 1960 for *A. coriophora*, 1600 for *A. laxiflora* and *H. robertianum*, and 320 for *H. hircinum*, resulting in 392, 320, 320, and 64 vessels, respectively (Table 1). Some vessels appeared to have more than five protocorms at the end of the experiment, likely a result of being attached together when transferred. Final protocorm counts per treatment are detailed in Supplementary Table S2.

### Experimental set-up

To ascertain whether temperature influences seedling development, the seedlings were subjected to different temperature treatments as follows: (i) ambient lab temperature of ∼18°C; (ii) refrigerated at ∼7°C; and (iii) natural temperature fluctuations at six outdoor stations along a steep altitudinal gradient (Table 2). The six outdoor stations were chosen based on the assumption that temperature decreases with altitude (Ackerman and Knox 2007). The vessels of each species were randomized and distributed equally among eight crates covered with a mesh of chicken wire and wrapped in plastic bags to protect them from rain and feral animals. The bags were semi-translucent to provide weak light exposure, as advised for young seedlings (S. Malmgren, personal communication, 2020). Inside the refrigerator, illumination was provided by cool white, fluorescent tubes (Osram Fluora L36W/77, Germany) with a 12-h photoperiod. At the outdoor stations, the crates were placed away from direct sunlight to avoid excessive warming due to any greenhouse effect. Temperatures were monitored at hourly intervals with miniaturized portable temperature loggers (Elitech®, Milpitas, CA, USA) enclosed in an empty culture vessel placed in the middle of each crate. In addition, two of the outdoor locations were equipped with weather stations. Every 10 days, the vessels were randomized within each crate so that they received equal amounts of light. The cold temperature treatment started on 25 January 2021 and lasted 8 weeks, until 23 March 2021. At the end of week 8, all crates were assembled back to Lakomatia, a station of intermediate altitude (492 m a.s.l.), to be gradually acclimatized to spring temperatures.

**Table 2:** Location and altitude above sea level of the temperature treatments with minimum, maximum, median, and mean ± SD of hourly temperature (°C), mean ± standard deviation (SD) of daily temperature range (°C), and cumulative hours below 6, 10, and 12°C (C_6_, C_10_, C_12_) inside the vessels during the 8 weeks of cold treatment.

Location	Coordinates	Altitude (m)	Min daily temp (°C)	Max daily temp (°C)	Median daily temp (°C)	Mean daily temp (°C) ± SD	Mean of daily range (°C) ± SD	C_6_	C_10_	C_12_
Refrigerator	34SDH5587824633	6	5.2	7.7	6.8	6.9 ± 0.7^a^	1.6 ± 0.9^a^	96	1351	1353
Laboratory	34SDH5587824633	6	16.0	20.8	18.3	18.4 ± 1.6^b^	3.7 ± 1.7^b^	0	0	0
Vlachata	34SDH6726319739	238	5.0	15.2	12.4	11.9 ± 3.2^c^	7.5 ± 1.9^c^	42	380	698
Lakomatia	34SDH6310023485	492	2.5	12.7	8.4	8.2 ± 3.1^d^	6.5 ± 2.3^d^	316	902	1210
Reservoir	34SDH6591928049	707	2.9	11.1	8.1	7.9 ± 2.7^e^	6.0 ± 2.5^e^	300	1085	1259
Road Bend	34SDH6754527061	916	0.4	10.3	7.0	6.7 ± 2.6^a^	4.4 ± 2.2^g^	457	1244	1333
Yupari	34SDH6622926477	1122	0.0	9.5	6.3	5.9 ± 3.0^f^	6.2 ± 2.5^e^	663	1245	1307
Ainos	34SDH6785422948	1291	1.2	9.8	5.8	5.9 ± 2.6^f^	4.1 ± 2.6^f^	703	1257	1335

Coordinates are in the Military Grid Reference System.

^[a–f]^Means with the same letter are not significantly different at *ɑ* = 0.05 (pairwise *t*-test).

### Measurements of plantlet growth

During the ensuing 8 weeks of acclimatization, shoot length was monitored with a ruler three times, i.e. at the end of 8, 12, and 16 weeks after the onset of cold treatment. The crates remained shaded at the Lakomatia station until the onset of summer dormancy, signalled by shoots starting to wither and tubers turning from white to cream colour. The crates were assembled back into the laboratory and tuber measurements started on 9 August 2021, 28 weeks after the onset of the cold treatment, as follows: The plantlets were extracted from the medium, washed in a 2-mm sieve under running water, and patted dry with paper towels. The length of the longest leaf was recorded. The height and width of each tuber were measured with a vernier calliper and used to approximate its volume (volume of ellipsoid = 4/3*πabc*, where *a*, *b*, and *c* are the radii along each axis). Tubers shorter than 1 mm were discarded as non-viable. The tubers from each vessel were weighed on a laboratory scale (precision ± 0.001 g), returned to their vessels, photographed, covered with slightly moistened soil, and stored loosely capped in a dark, non-air-conditioned room to simulate summer dormancy conditions in nature.

By 23 November 2021, 43 weeks after the onset of the cold treatment, the indoor environment had substantially cooled. The end of summer dormancy was signalled by most tubers sprouting a new shoot. The plantlets were extracted from the soil, washed with a thin jet of deionized water in a 2-mm sieve, and patted dry with paper towel (Fig. 1i). The length of each plantlet (tuber plus shoot) and its mass were recorded.

### Statistical analyses

#### Temperature

Temperature was plotted throughout the cold treatment and subsequent acclimatization period. For each station, minimum, maximum, median, and mean temperature, temperature range, and cumulative hours below 13 temperature thresholds (<0°C to <12°C) were calculated to be used as explanatory variables in the statistical analyses. The thresholds were chosen according to the range of temperatures reported in the literature to relieve protocorm dormancy (2°C–12°C, in Hadley 1970, Beyrle et al. 1985, Ponert et al. 2012, Malmgren and Nyström 2026), plus the thresholds of 0°C and 1°C that are believed harmful to plant tissues (Pfeifer et al. 2006). Linear regression was used to explore the relationship between the cumulative hours below selected temperature thresholds and altitude. Mean temperatures and mean daily temperature fluctuations at the eight stations were compared with paired *t*-tests.

#### Survival assessment

Because individual plantlets could not be tracked across developmental stages, pre-dormancy survival was inferred from functional thresholds linking below-ground reserve accumulation to above-ground growth. The use of tuber volume thresholds was preferred over visual assessment of shoot browning (commonly used to assess survival; Van Waes 1984), as some individuals classified with browned shoots still possessed viable tubers (data not shown). For each species, the threshold was determined empirically by identifying the smallest tuber volume from which at least two individuals produced a functional shoot, defined as reaching at least 10% of the maximum shoot length observed for that species. This criterion was chosen to avoid defining the threshold from isolated individuals that could represent measurement error or atypical development. Although empirical, this approach provided a conservative criterion aimed at minimizing false classification of living individuals as dead. The resulting species-specific tuber volume thresholds (2.0, 1.0, 2.0, and 10.5 mm^3^ for *A. coriophora*, *A. laxiflora*, *H. robertianum*, and *H. hircinum*, respectively) corresponded to minimum shoot lengths of 14, 12.5, 6.5, and 8.3 mm. Individuals with tuber volumes below these thresholds were considered non-viable prior to dormancy. Post-dormancy survival was directly assessed depending on the emergence of a shoot.

#### Plant performance

To identify which components of thermal regime best explained variation in plant performance, we used an information-theoretic model selection approach (Burnham and Anderson 2002). We used the five continuous descriptors of the thermal regime experienced during the 8 weeks of cold treatment (minimum, maximum, median, mean temperature, temperature range) and the 13 cumulative chilling indices (C_0_–C_12_), where C*_x_* represents the number of hours with temperature below *x*°C. These variables capture both the intensity and duration of cold exposure. For each response variable (pre-dormancy and post-dormancy survival; pre-dormancy shoot length, tuber volume, and tuber mass per vessel; post-dormancy tuber mass and shoot length), we fitted a set of competing generalized linear or linear models in which each thermal variable was entered separately, together with species identity and their interaction, allowing for species-specific thermal responses (R package ‘lme4’; Bates et al. 2015). For each thermal predictor, we fitted both linear and quadratic equations to account for monotonic effects as well as non-linear (optimum-type) physiological responses. Binomial error distributions were used for survival models, and Gaussian errors were used for continuous traits after appropriate variance-stabilizing transformations (see Supplementary Tables for details). All models were compared using AICc (Akaike Information Criterion corrected for small sample size) as implemented in the MuMIn package (Burnham and Anderson 2002). For each trait, we ranked all candidate models and identified the most parsimonious predictors based on ΔAICc and Akaike weights. This procedure allowed us to determine which thermal thresholds and temperature descriptors best explained each biological process, and whether responses were best described by linear or non-linear relationships. When a given predictor was strongly supported by model selection, we refitted the corresponding model and its species-specific components to estimate coefficients and visualize thermal response curves. All analyses were performed in R version 4.1.3 (R Core Team 2021).

## Results

### Temperature

Mean temperature at the outdoors stations varied from 11.9 ± 3.2°C (mean ± SD) in Vlachata to 5.9 ± 2.6°C in Ainos (238 and 1291 m a.s.l., respectively; Table 2), consistent with temperature decreasing with altitude (Ackerman and Knox 2007). Laboratory was the warmest with mean temperature of 18.4 ± 1.6°C. Except for Yupari and Ainos (5.9 ± 3.0°C and 5.9 ± 2.6°C, respectively), mean temperatures were significantly different among stations (Table 2). Refrigerator, Laboratory, and Ainos stations experienced the lowest mean daily fluctuations of 1.6 ± 0.9°C, 3.7 ± 1.7°C, and 4.1 ± 2.6°C, respectively, while the highest fluctuations were at Vlachata, Lakomatia, and Yupari stations, of 7.5 ± 1.9°C, 6.5 ± 2.3°C, and 6.2 ± 2.5°C, respectively (Table 2). The total number of hours below a temperature threshold throughout the cold treatment increased with altitude following a linear relationship (Supplementary Figure S1). Laboratory, with a minimum temperature of 14°C, was the only station without any hours below the temperature thresholds. During the 8 weeks of the acclimatization period at Lakomatia, hourly temperatures ranged between 2.8°C and 27.7°C with a mean of 14.1 ± 4.8°C.

Temperature was likely the main factor influencing orchid growth in our study. Although light effects cannot be entirely excluded, all outdoor stations experienced the same natural photoperiod, were located in close geographical proximity, and seedlings were maintained in identical semi-translucent bags protected from direct sunlight, resulting in similar daylength and broadly comparable illumination. In contrast, the refrigerated treatment was excluded from subsequent analyses because plantlets produced outlier responses, likely resulting from exposure to nearly constant temperature and photoperiod (Stewart and Kane 2006, Calevo and Bazzicalupo 2020, Nabieva et al. 2025), precluding direct comparison with the outdoor treatments.

### Plant survival

**Figure 2:**
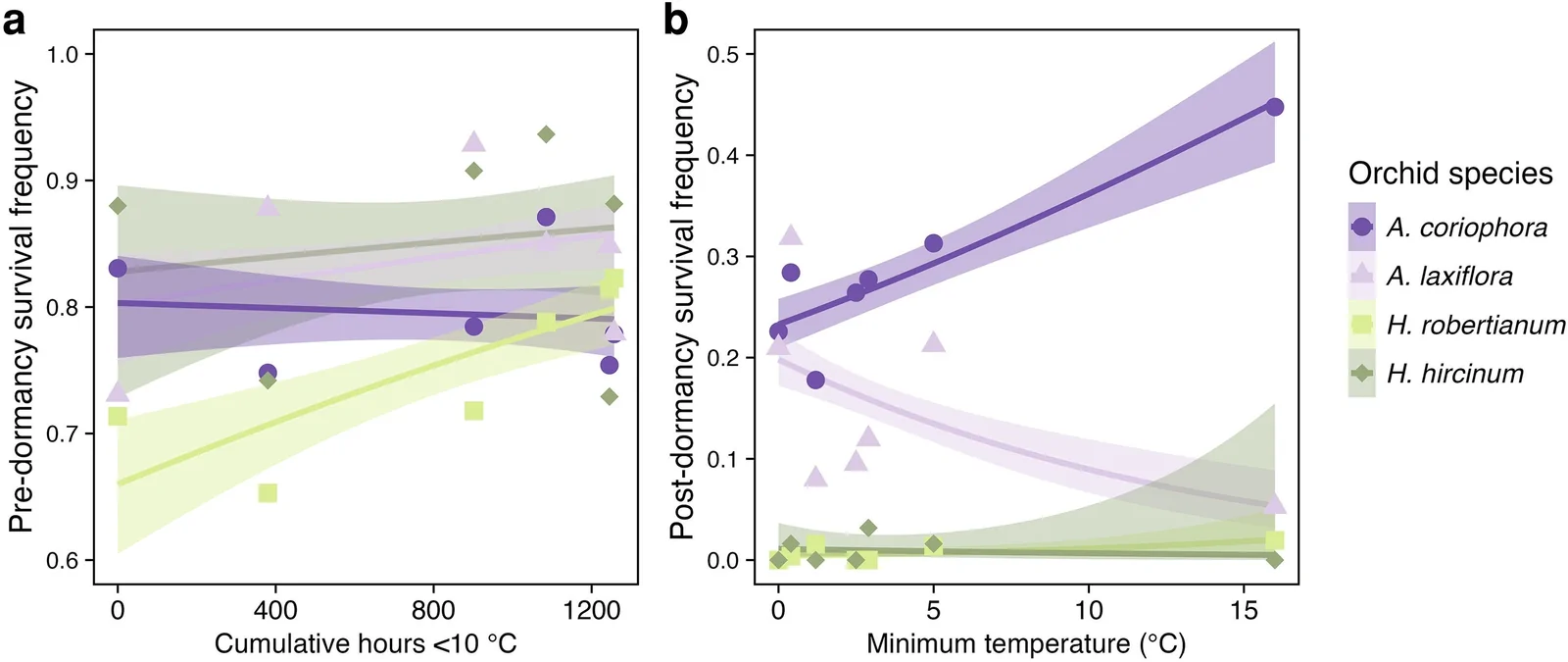
Effect of cumulative hours below 10°C on pre-dormancy survival (a) and minimum temperature (°C) on post-dormancy survival (b) of *Anacamptis coriophora*, *A. laxiflora*, *Himantoglossum robertianum*, and *H. hircinum* (sample sizes in Supplementary Table S2). Solid lines are logistic regression for each species with 95% confidence intervals. Chilling increased pre-dormancy survival in *A. laxiflora* and *H. robertianum*, while no significant effect was detected in *A. coriophora* or *H. hircinum*. Low temperature reduced survival in *A. coriophora* and *H. robertianum*, increased survival in *A. laxiflora* but had no effect on *H. hircinum* (see Supplementary Table S4 for details).

Overall, pre-dormancy survival was high and differed among species (*P* < 0.0001; Supplementary Table S4a) averaging (mean ± SE) 80.6 ± 0.9%, 84.3 ± 0.9%, 77.4 ± 1.0%, and 85.2 ± 1.7% for *A. coriophora*, *A. laxiflora*, *H. robertianum*, and *H. hircinum*, respectively. Survival was primarily controlled by cumulative exposure to moderately low temperature rather than by extreme cold. Model selection identified cumulative hours below 10°C–11°C (C_10_–C_11_) as the best predictor of survival (*P* < 0.0001; Supplementary Table S3). Survival increased significantly with increasing C_10_ in *A. laxiflora* and *H. robertianum* (*P* = 0.0242 and *P* < 0.0001, respectively), while no significant effect was detected in *A. coriophora* or *H. hircinum* (*P* = 0.64 and *P* = 0.51, respectively; Fig. 2a, Supplementary Table S4a). Accordingly, the global model showed a significant C_10_ × species interaction (*P* = 0.0058), indicating divergent chilling sensitivities among taxa.

Post-summer dormancy survival differed strongly among species (*P* < 0.0001; Supplementary Table S4b). While survival was still relatively high for *Anacamptis* species (28.4 ± 1.0% and 15.6 ± 0.9% for *A. coriophora* and *A. laxiflora*, respectively), it was close to zero for *Himantoglossum* species (0.7 ± 0.2% and 0.9 ± 0.5% for *H. robertianum* and *H. hircinum*, respectively). Post-dormancy survival was best explained by minimum temperature experienced during the cold treatment rather than by cumulative chilling (Supplementary Table S3). Species differed strongly in their responses (interaction min temperature × species, *P* < 0.0001; Fig. 2b, Supplementary Table S4b): low temperature reduced survival in *A. coriophora* and *H. robertianum* (*P* < 0.0001 and *P* = 0.0240, respectively), increased survival in *A. laxiflora* (*P* < 0.0001) but had no effect on *H. hircinum* (*P* = 0.71). The thermal stress induced by cold treatment impacted species in contrasting ways even under identical post-dormancy growing conditions.

### Pre-dormancy shoot growth

Shoot growth during the acclimatization phase (post cold treatment) was strongly temperature dependent (*P* < 0.0001) and differed among species (*P* < 0.0001). Overall, *A. laxiflora* had the highest growth rate, growing from a mean shoot length of 8.6 ± 0.3 to 44.5 ± 0.7 mm (growth rate = 517.4%), followed by *A. coriophora*, growing from 21.8 ± 0.5 to 54.8 ± 0.7 mm (growth rate = 251.4%). *Himantoglossum* species had the lowest growth rates: *H. hircinum* shoots grew from 6.2 ± 0.4 to 11.0 ± 0.8 mm (growth rate = 177.4%) and *H. robertianum* from 2.6 ± 0.4 to 3.7 ± 0.1 mm (growth rate = 142.3%). Across all weeks, growth was best described by quadratic functions of median temperature, with highly significant interactions between temperature, species, and time (*P* < 0.0001). This indicates that shoot growth followed species-specific thermal reaction norms rather than monotonic responses to cold.

**Figure 3:**
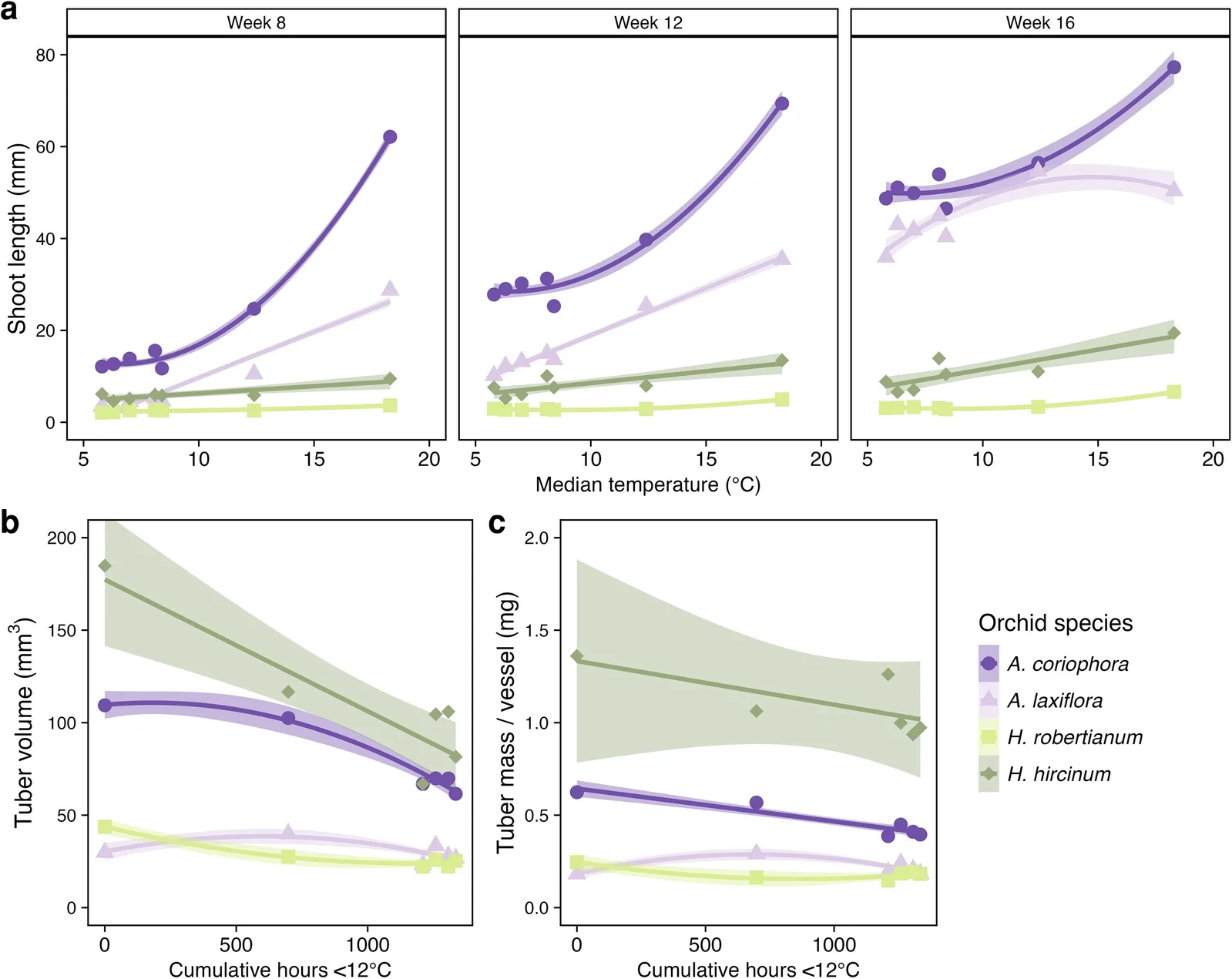
Effect of median temperature on pre-dormancy shoot growth (a) at 8, 12, and 16 weeks, and of cumulative hours below 12°C on pre-dormancy tuber volume (b) and tuber mass (c) per vessel of *Anacamptis coriophora*, *A. laxiflora*, *Himantoglossum robertianum*, and *H. hircinum* (sample sizes in Supplementary Table S2). Solid lines are regressions for each species with 95% confidence intervals. Shoot length (mm) measured from the medium surface to the tip of the longest leaf. Warmer temperature during the cold treatment generally induced a faster shoot growth even though the response differed between species, while chilling generally induced a reduction in tuber volume and mass that was more pronounced for *A. coriophora* and *H. hircinum* (see Supplementary Tables S5 and S6 for details).

By the end of week 8, growth increased strongly with temperature in all species (*P* < 0.0001), but the shape of the response differed (interaction median temperature × species: *P* < 0.0001). *Anacamptis coriophora* showed a steep non-linear response, whereas *A. laxiflora*, *H. robertianum*, and *H. hircinum* responded mainly linearly (Supplementary Table S5a, Fig. 3a). By the end of week 16, growth accelerated and non-linear responses were observed for all species except *H. hircinum* (Supplementary Table S5b and c, Fig. 3a). For *Anacamptis* species, the shoot length difference between treatments was less pronounced in week 16 than in week 8 (Fig. 3a), indicating that cold treatment initially slowed growth and that species differed in their capacity to compensate during the acclimatization period.

### Pre-dormancy tuber growth

At the onset of summer dormancy, tubers of *H. hircinum* were the largest, with mean volume of 99.3 ± 6.6 mm^3^ (mean ± SE) and mean mass per vessel of 1.060 ± 0.107 g; tubers of *A. coriophora* were of intermediate size, with mean volume of 76.0 ± 1.5 mm^3^ and mean mass per vessel of 0.464 ± 0.010 g and tubers of *A. laxiflora* and *H. robertianum* were the smallest, with mean volume of 30.3 ± 0.8 and 26.7 ± 0.8 mm^3^ and mean mass per vessel of 0.217 ± 0.006 and 0.192 ± 0.009 g, respectively (*P* < 0.0001; Supplementary Table S4a and b).

Tuber volume (*P* < 0.0001) and mass (*P* < 0.0001) before dormancy were primarily controlled by cumulative exposure to moderate cold. For both traits, cumulative hours below 12°C (C_12_) provided the best fit (Supplementary Table S3), with strong quadratic effects and significant species interactions (*P* < 0.0001 for both traits; Supplementary Table S6a and b). Cold generally induced a reduction in tuber volume and mass that was more pronounced for *A. coriophora* and *H. hircinum*, with a reduction of 43.8% and 55.9% in volume and 36.7% and 28.6% in mass, respectively (Fig. 3b and c). In *A. coriophora*, *A. laxiflora*, and *H. robertianum*, tuber volume and mass followed non-linear response curves, indicating an optimal range of chilling for reserve accumulation (Fig. 3b and c). In contrast, *H. hircinum* exhibited only weak or linear responses with a decrease of tuber volume (*P* < 0.0001) and mass (*P* = 0.0985) induced by the accumulation of chilling (Fig. 3b and c; Supplementary Table S6a and b).

### Post-dormancy growth

**Figure 4:**
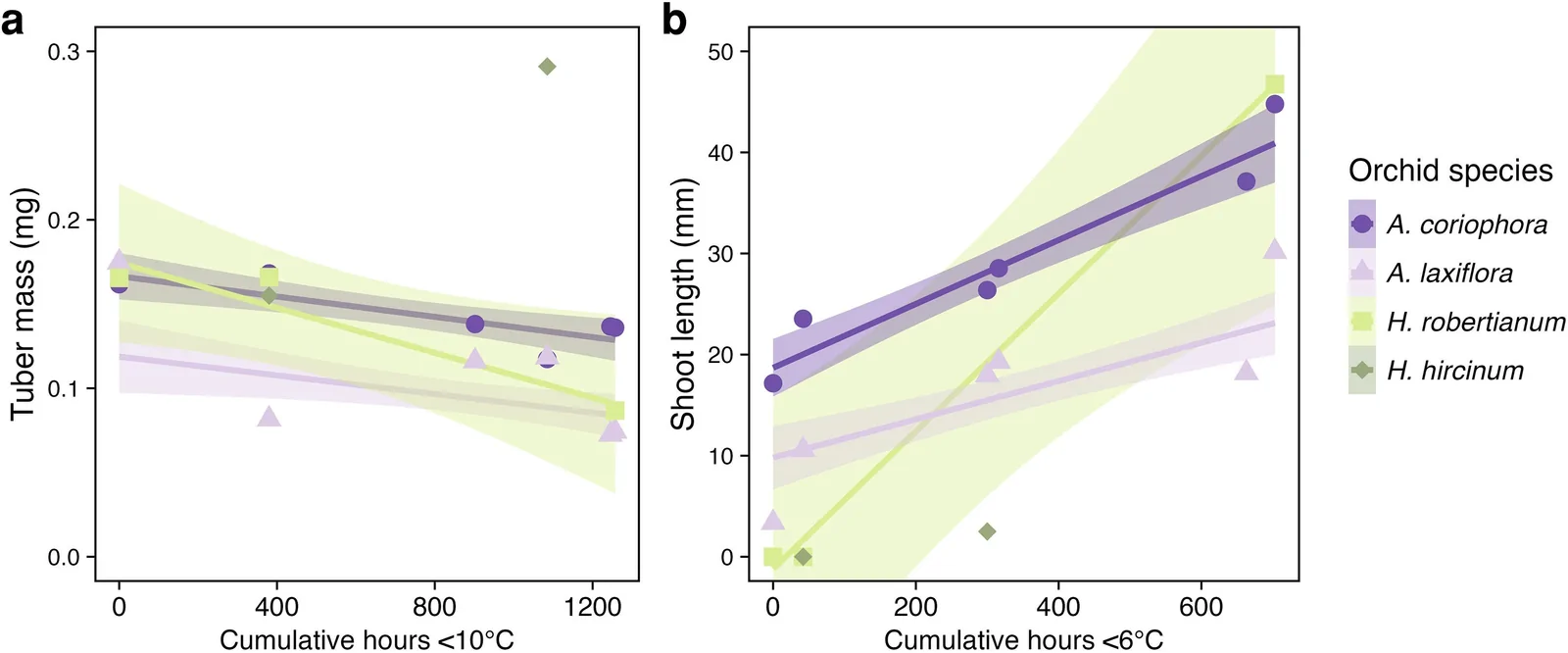
Effect of cumulative hours below 10°C on post-dormancy tuber mass (a) and cumulative hours below 6°C on post-dormancy shoot length (b) of *Anacamptis coriophora*, *A. laxiflora*, *Himantoglossum robertianum*, and *H. hircinum* (sample sizes in Supplementary Table S2). Solid lines are linear regression curves for each species with 95% confidence intervals. While chilling negatively impacted tuber mass of *A. coriophora* and *A. laxiflora*, it strongly and positively impacted shoot growth in all species, except *H. hircinum* for which the relationship could not be assessed due to low sample size (see Supplementary Table S7 for details).

Post-dormancy, tuber mass was best predicted by cumulative hours below 10°C (C_10_; Supplementary Table S3). In *A. coriophora*, *A. laxiflora*, and *H. robertianum*, tuber mass linearly decreased with increasing C_10_ (*P* = 0.0004, *P* = 0.0002, and *P* = 0.0097, respectively), while in *H. hircinum*, there were insufficient survivors to assess any relationship (Supplementary Table S7a; Fig. 4a). In contrast, shoot growth responded strongly and positively to chilling. Shoot length increased linearly with C_6_ in *A. coriophora*, *A. laxiflora*, and *H. robertianum* (*P* < 0.0001 for all species), with intensity differing between species (interaction C_6_ × species: *P* = 0.0025; Supplementary Table S7b; Fig. 4b). *Himantoglossum robertianum* showed the most extreme response, with little or no shoot development under warm conditions but strong leaf emergence after prolonged chilling (*P* < 0.0001). *Himantoglossum hircinum* showed no detectable response (*P* = 0.28), mostly due to very low survival (Fig. 4b).

## Discussion

The effect of cold on orchid growth has received limited attention (Hadley 1970, Beyrle et al. 1985, Rasmussen 1995, Malmgren 2004; Kauth et al. 2008, 2011, Ponert et al. 2012, Malmgren and Nyström 2026). Nevertheless, several studies have suggested that exposure to low temperatures promotes the transition of orchid protocorms to plantlets, although the temperatures reported to be effective vary considerably among species and studies. We exposed protocorms of *Anacamptis* and *Himantoglossum* species to different thermal regimes and showed that plant responses were best explained by cumulative chilling exposure rather than by mean temperature alone, with species-specific responses and distinct thermal thresholds controlling different developmental processes.

### Survival response to cold is species-specific

Pre- and post-dormancy survival following cold treatment differed between species, likely due to differences in cold tolerance (Garbisch et al. 1995, Rasmussen 1995, Delforge 2006). Pre-dormancy survival was high overall (>60%) and, when chilling had an effect, it increased survival. Moderate winter chilling seems therefore to improve physiological preparedness for dormancy for some species without inducing physiological stress in other species. On the contrary, post-dormancy survival was drastically reduced in all species, dropping to <50% regardless of temperature treatment. Dormancy can induce increased mortality in some orchids, including *Neotinea ustulata* (L.) R.M. Bateman, Pridgeon & M.W. Chase, *Cypripedium parviflorum* Sims, *Cleistes bifaria* (Fernald) Catling & Gregg, and *Ophrys sphegodes* Mill. (Hutchings 1987, Shefferson et al. 2003, Gregg and Kéry 2006, Shefferson and Tali 2007). Post-dormancy survival of *Himantoglossum* species was extremely low, even though they are considered cold-hardy (Garbisch et al. 1995, Kauth et al. 2011). Their shoots also remained relatively small compared to those of *Anacamptis* species. *Anacamptis* species were more advanced in development at the onset of the experiment compared to *Himantoglossum* species (they had developed the first leaf while *Himantoglossum* were still at the protomeristem stage; stage 5 vs stage 4, according to Swarts and Dixon 2017). The smaller protocorms of *Himantoglossum* species might have not been ready to be exposed to cold, explaining the difference in post-dormancy survival. In *Anacamptis* species, cold induced either an increase (*A. coriophora*) or a decrease in survival (*A. laxiflora*), indicating strong differences in species sensitivity to winter thermal stress. Overall, the results indicate that seedling survival was not controlled by exposure to extreme cold, but by species-specific responses to winter thermal regimes. Moderate chilling can enhance survival in some taxa, while low minimum temperatures may become detrimental or beneficial depending on species.

### Cold slows pre-dormancy orchid growth but has a carry-over effect post-dormancy

Cold stress, including chilling (0°C–15°C) and freezing (<0°C), restricts growth, development, and geographical distribution of plants (Liu et al. 2018, Ding et al. 2020). Accordingly, in this study, pre-dormancy shoot and tuber growth were strongly controlled by temperature; all species exhibited non-linear responses to temperature, with mostly reduced shoot length and tuber size at low temperatures and species-specific responses. Although low temperature can hinder plant growth, a longer growing period can compensate for the decline in growth rate (Zhang et al. 2018). This was most evident in *Anacamptis* species, for which the size gap in shoot length between cold-treated and control plantlets decreased with time. Stabilization of tuber volume and mass is consistent with shoot growth eventually catching up. *Anacamptis* species also grew much longer shoots than *Himantoglossum* species, likely due to differences in growth rates (Poorter 1989), although the small size of *Himantoglossum* tuber protocorms at the onset of the experiment could also explain the difference in final shoot length (see Survival response to cold is species-specific section above). Control plantlets in the laboratory also grew faster and taller than cold-treated ones, allowing them to start photosynthesis earlier and therefore develop storage organs faster (Tissue et al. 1995). Growing larger tubers more rapidly ensures the development of storage organs during shortened growing season and increases winter survival, as reported for *Calopogon tuberosus* (L.) Britton, Sterns & Poggenb. (Kauth et al. 2008).

Some studies suggest an optimum temperature for orchid growth, at which the rate of progress towards a particular developmental event is maximal (e.g. Zhang et al. 2018). This optimal temperature was apparent in *A. laxiflora* shoot growth, which was optimized at an intermediate median temperature of 14°C, and in tuber volume and mass of *A. coriophora* and *A. laxiflora*, which were maximized at intermediate cumulative hours of cold, indicating an optimal chilling range for reserve accumulation. The optimal temperature, and the requirement of cold treatment for growth, while species-specific, is also likely to be influenced by the environment from which the seeds originate (Rasmussen et al. 2015). For instance, northern populations of *C. tuberosus* require a minimum chilling period of 8 weeks for maximum shoot emergence, likely due to the experience of a longer winter in the field compared to southern plants (Kauth et al. 2011). Similarly, most studies reporting benefit of cold treatment on orchid growth used seeds of specimens originating from cold climates (Hadley 1970, Beyrle et al. 1985, Ponert et al. 2012, Malmgren and Nyström 2026). However, contrary to our expectation that *H. hircinum* seeds would benefit more from a cold treatment than seeds from the other species that were harvested from lower latitudes in the thermomediterranean climatic zone of Cephalonia, cold impaired growth and development of this species. This result was consistent with a 26-year survey of *H. hircinum* populations in a natural reserve in Germany, where colder winter conditions reduced survival and growth rates of the plants (Pfeifer et al. 2006). It is therefore likely that the differences between species seen in our study reflect species-specific cold tolerance and requirements rather than geographical origin. Future research should compare the response of orchid species to cold treatment along latitudinal gradients to better decipher species versus local environment effects.

The beneficial effect of cold treatment was mostly visible post-dormancy, revealing a carry-over effect on growth (O'Connor et al. 2014). In other words, thermal conditions experienced before dormancy continued to influence plant performance during the following growing season through persistent physiological changes, notably reserve accumulation. Shoot length increased steeply with cumulative exposure below 6°C–7°C in *A. coriophora*, *A. laxiflora*, and *H. robertianum*, and only cold-exposed plantlets of *H. robertianum* and *H. hircinum* grew leaves. This demonstrates that chilling does not suppress growth but, rather, promotes subsequent shoot development in most species, consistent with a physiological chilling requirement for release of dormancy and efficient mobilization of reserves. Post-dormancy, tuber mass declined with increasing cumulative exposure below 10°C in *A. coriophora* and *A. laxiflora*, indicating that stronger chilling led to greater mobilization of reserves for shoot growth. Together, the increase of shoot growth and decline in tuber mass indicate that cold exposure promotes translocation of stored carbon into above-ground growth rather than directly increasing tuber size. The increase of leaf surface will subsequently influence the volume and mass of the tubers, as tuber storage (and therefore size) is a result of photosynthesis (Tissue et al. 1995). It is likely that cold-exposed tubers enter a positive loop where a longer shoot will promote higher carbon storage leading to longer shoots during the following season. Together, these results indicate that chilling primarily enhances carbon storage before dormancy and stimulates its mobilization for shoot growth after dormancy. The positive effects of cold treatment are therefore expressed most clearly in the following growing season, rather than during the chilling period itself.

## Conclusions

This study focused on the influence of temperature on the growth of temperate terrestrial orchids cultivated *in vitro*. Our data demonstrate that cold temperatures regulate growth through species-specific, non-linear physiological responses rather than exposure to extreme cold. Cumulative exposure to moderately low temperatures (10°C–12°C) controls survival and reserve accumulation before dormancy, while chilling below 6°C–7°C promotes post-dormancy shoot growth by enhancing reserve mobilization. These carry-over effects generate positive feedback between winter chilling and growth during the following season. Importantly, thermal responses differed strongly among species, indicating that chilling requirements reflect species-specific physiological strategies. Besides their relevance for *in vitro* culture, these results suggest that warmer winters may disrupt both survival and post-dormancy development, with consequences that vary among taxa. In natural populations, orchid growth also depends on associations with mycorrhizal fungi, whose seasonal dynamics may further influence carbon acquisition and storage in response to winter temperatures (Calevo and Duffy 2023). Understanding how temperature and mycorrhizal interactions jointly shape orchid performance will therefore be important for conservation, restoration, and *ex situ* propagation of orchids in the face of ongoing climate change.

## Supplementary Material

plag039_Supplementary_Data

## Data Availability

The data supporting the results and the code used are freely available on Zenodo.org (https://doi.org/10.5281/zenodo.22030723). The DOI represents all versions and will always resolve to the latest one.
