# Supplementary material for: Winter chilling regulates seedling development in Mediterranean terrestrial orchids cultivated in vitro
Source: AoB Plants. 2026 Aug 27;18(5):plag039. doi: 10.1093/aobpla/plag039 (PMC13557010; doi:10.1093/aobpla/plag039)
Supplement: Supplementary file 1 [file plag039_supplementary_data.pdf]

Winter chilling regulates seedling development in Mediterranean terrestrial orchids cultivated *in vitro*

Gwenaëlle Deconninck[1,2*], Argyrios Gerakis[1], Victoria Chatzopoulou[1], Alexandra Thoma[3], Eirini Katsalirou[1]

[1]Department of Food Science and Technology, Ionian University, Greece

[2]Department of Biology, Lund University, Lund, Sweden

[3]Università degli Studi della Basilicata, Potenza, Italy

[*]Corresponding author: gwenaelle.deconninck@biol.lu.se

Running title: Cold regulates terrestrial orchid growth

Supporting information

Table S1. Composition of modified "SM-organic" nutrient medium.

Table S2. Sample sizes (Refrigerator treatment excluded).

Table S3. Model selection for various traits.

Table S4. Logistic regression models of the influence of cumulative hours below 10 °C and minimum temperature on survival.

Table S5. Linear regression models of the effect of median temperature on pre-dormancy shoot length after the onset of cold treatment.

Table S6. Linear regression models of the effect of cumulative hours below 12 °C on pre-dormancy tuber volume and tuber mass per vessel.

Table S7. Linear regression models of the effect of cumulative hours below 10 °C and 6 °C on post-dormancy tuber mass and shoot length.

Figure S1. Cumulative hours below a temperature threshold at the end of the cold treatment as a function of altitude above sea level.

Table S1. Composition of modified “SM-organic” nutrient medium.

**Ingredients**	**Quantity**
Mineral water	1 L
Saccharose (sucrose)	12.5 g
KH_2PO_4	75 mg
$MgSO_4 \cdot 7H_2O$	150 mg
$CaHPO_4$	100 mg*
H_3BO_3	10 mg
Activated charcoal	750 mg
Danish agar	10 g
Vaminolac® (Fresenius Kabi, Uppsala, Sweden)	4.6 mL
Pineapple juice	20 mL
3 M H_2SO_4 for pH adjustment	~ 10 drops
Potato (*Solanum tuberosum*) tuber	1 cm^3 per culture vessel

*Equivalent to 75 mg $Ca_3(PO_4)_2$ in the original formula

Table S2. Sample sizes (Refrigerator treatment excluded).

Species	Number of hours <6°C	Initial number of protocorms	Pre-dormancy: Shoot length	Pre-dormancy: Tuber volume	Pre-dormancy: Tuber mass per vessel	Pre-dormancy: Survivors	Post-dormancy: Survivors, tuber mass and shoot length
A. coriophora	0	248	245	206	48	206	111
	42	246	245	184	45	184	77
	300	256	245	224	48	223	71
	316	246	245	194	49	193	65
	457	257	245	223	49	223	73
	663	248	245	187	48	187	56
	703	253	245	197	47	197	45
A. laxiflora	0	208	245	152	38	152	11
	42	221	245	195	38	194	47
	300	226	245	193	37	192	27
	316	252	245	234	40	234	24
	457	223	245	193	38	193	71
	663	224	245	190	37	190	47
	703	213	245	166	38	166	17
H. robertianum	0	206	200	154	38	147	4
	42	219	200	155	37	143	3
	300	250	200	211	39	197	0
	316	234	200	184	38	168	0
	457	263	200	232	39	229	1
	663	253	200	213	40	206	0
	703	254	200	214	38	209	4
H. hircinum	0	50	40	46	8	44	0
	42	62	40	52	7	46	1

300	63	40	63	8	59	2
316	65	40	64	8	59	0
457	62	40	62	8	53	1
663	48	40	37	6	35	0
703	76	40	75	8	67	0

Table S3. Model selection for various traits. Models retained for analyses are highlighted in bold.

Trait	Model	AICc	delta	weight
**Survival pre-dormancy**	**C_{10}^2**	**5187.8**	**0**	**0**
	C_{11}^2	5187.9	0	0
	C_9^2	5192.1	4.2	4.2
	$Temp\_fluc^2$	5192.6	4.8	4.8
	C_{12}^2	5193.9	6	6
**Growth**	**$Temp\_med^2$**	**38217.3**	**0**	**0**
	$Temp\_mean^2$	38226.7	9.5	9.5
	C_{12}^2	38239.3	22.1	22.1
	C_{11}^2	38246	28.7	28.7
	C_{10}^2	38260.1	42.9	42.9
**Tuber mass pre-dormancy**	**$C12^2$**	**-799.2**	**0**	**0**
	$Temp\_med^2$	-796.8	2.3	2.3
	$Temp\_mean^2$	-796.7	2.5	2.5
	C_{11}^2	-796.5	2.6	2.6
	C_{10}^2	-793.7	5.5	5.5
**Tuber volume pre-dormancy**	**C_{12}^2**	**12815.7**	**0**	**0**
	C_{11}^2	12827.9	12.2	12.2
	$Temp\_med^2$	12838.5	22.7	22.7
	C_{10}^2	12839.8	24	24
	$Temp\_mean^2$	12840.5	24.8	24.8
**Survival post-dormancy**	**Temp_min**	**3579**	**0**	**0**
	$Temp\_min^2$	3584.4	5.4	5.4
	$Temp\_med^2$	3587.1	8.2	8.2
	Temp_max	3587.7	8.8	8.8
	Temp_mean	3589	10	10
**Shoot length post-dormancy**	**C_6**	**3176.6**	**0**	**0**
	C_7	3177.1	0.6	0.6

	Temp_med[2]	3178.9	2.3	2.3
	C_5	3179.6	3	3
	C_8	3180.5	4	4
**Tuber mass post-dormancy**	Temp_max	-1247.8	0	0
	**C_{10}**	**-1246.6**	**1.2**	**1.2**
	Temp_min	-1246.5	1.3	1.3
	C_{11}	-1246.5	1.3	1.3
	Temp_mean	-1246.1	1.7	1.7

Abbreviations: C_0–C_{12}: cumulative chilling indices, where C_x represents the number of hours with temperature below x °C; Temp_min, Temp_max, Temp_mean and Temp_med: minimum, maximum, mean and median temperature; Temp_fluc: mean of daily temperature range.

Table S4. Logistic regression models of the influence of cumulative hours below 10 °C (C_{10}) on pre-dormancy survival (A) and minimum temperature (Temp_min) on post-dormancy survival (B) of *Anacamptis coriophora*, *A. laxiflora*, *Himantoglossum robertianum* and *H. hircinum* (sample size in Table S2).

	**Intercept**	**Slope**	**SE**	***z*-value**	***p*-value**		**Effect**
**(A) Effect of cumulative hours below 10 °C on pre-dormancy survival**							
**Global model**							
C_{10}					**1.88e-06**	***	Overall C_{10} effect
Species					**5.26e-07**	***	Species differences
C_{10}:Species					**0.0058**	**	Species-specific response
**Species model**							
A. coriophora	1.369	0.00006	0.00013	0.46	0.6429		No chilling effect
A. laxiflora	1.394	0.00033	0.00015	2.25	**0.0242**	**	Positive chilling effect
H. robertianum	0.629	0.00069	0.00012	5.52	**3.42e-08**	***	Strong positive chilling effect
H. hircinum	1.576	0.00020	0.00030	0.66	0.5125		No chilling effect
**(B) Effect of minimum temperature (°C) on post-dormancy survival**							
**Global model**							
Temp_min					**0.0083**	**	Overall cold temperature effect
Species					**<2e-16**	***	Species differences
Temp_min:Species					**2.68e-14**	***	Species-specific cold response
**Species model**							
A. coriophora	-1.193	0.063	0.010	6.45	**1.12e-10**	***	Lower temperature reduces survival

A. laxiflora	-1.399	-0.092	0.020	-4.62	**3.86e-06**	***	Lower temperature increases survival
H. robertianum	-5.435	0.098	0.043	2.26	**0.0240**	*	Lower temperature reduces survival
H. hircinum	-4.499	-0.050	0.134	-0.37	0.7105		No temperature effect

Table S5. Linear regression models of the effect of median temperature (Temp_med) on pre-dormancy shoot length in mm of *Anacamptis coriophora, A. laxiflora, Himantoglossum robertianum* and *H. hircinum* at the end of 8 (A), 12 (B), and 16 (C) weeks after the onset of cold treatment (sample size in Table S2).

Species	Adj-R^2	Intercept	Lin term	Lin SE	Quad term	Quad SE	Lin *p*-value	Quad *p*-value	Effect
**(A) Effect of median temperature on pre-dormancy shoot length - Week 8**									
**Global model**									
Temp_med^2								**<2e-16** ***	Strong nonlinear thermal control
Species								**<2e-16** ***	Strong species differences
Temp_med^2:Species								**<2e-16** ***	Species-specific thermal response
**Species model**									
A. coriophora	0.49	4.165	59.644	1.504	15.499	1.502	**<2e-16** ***	**<2e-16** ***	Strong accelerating response
A. laxiflora	0.47	0.510	0.140	0.004	NA	NA	**<2e-16** ***		Strong linear thermal response
H. robertianum	0.07	1.014	0.022	0.002	NA	NA	**<2e-16** ***		Weak linear response
H. hircinum	0.04	1.520	0.028	0.008	NA	NA	**2.3e-04** ***		Weak linear response
**(B) Effect of median temperature on pre-dormancy shoot length - Week 12**									
**Global model**									
Temp_med^2								**<2e-16** ***	Strong nonlinear thermal control
Species								**<2e-16** ***	Strong species differences
Temp_med^2:Species								**<2e-16** ***	Species-specific thermal response

**Species model**											
A. coriophora	0.21	5.509	44.296	2.131	12.165	2.128	**<2e-16**	***	**1.3e-08**	***	Strong optimum-type response
A. laxiflora	0.26	1.735	0.221	0.010	NA	NA	**<2e-16**	***			Linear thermal response
H. robertianum	0.07	0.919	5.832	0.627	2.847	0.638	**<2e-16**	***	**8.9e-06**	***	Optimum-type response
H. hircinum	0.07	1.365	0.0468	0.010	NA	NA	**7.0e-06**	***			Linear thermal response
**(C) Effect of median temperature on pre-dormancy shoot length - Week 16**											
**Global model**											
Temp_med^2									**<2e-16**	***	Strong nonlinear thermal control
Species									**<2e-16**	***	Strong species differences
Temp_med^2:Species									**<2e-16**	***	Species-specific thermal response
**Species model**											
A. coriophora	0.10	54.785	375.094	28.893	116.501	28.854	**<2e-16**	***	**5.6e-05**	***	Strong accelerating response
A. laxiflora	0.03	44.440	172.335	27.690	-106.561	28.185	**6.4e-10**	***	**1.6e-04**	***	Clear thermal optimum
H. robertianum	0.08	1.735	7.687	0.772	3.658	0.786	**<2e-16**	***	**3.6e-06**	***	Optimum-type response
H. hircinum	0.07	1.373	0.059	0.013	NA	NA	**5.8e-06**	***			Linear thermal response

Table S6. Linear regression models of the effect of cumulative hours below 12 °C (C_{12}) on pre-dormancy tuber volume in mm^3 (A) and tuber mass per vessel in g (B) of *Anacamptis coriophora, A. laxiflora, Himantoglossum robertianum* and *H. hircinum* (sample size in Table S2).

Species	$Adj\text{-}R^2$	Intercept	Lin term	Lin SE	Quad term	Quad SE	Lin *p*-value	Sig.	Quad *p*-value	Sig.	Effect
**(A) Effect of cumulative hours below 12 °C on pre-dormancy tuber volume**											
**Global model**											
C_{12}^2									**3.3e-16**	***	Strong nonlinear chilling effect
Species									**<2e-16**	***	Strong species differences
C_{12}^2:Species									**<2e-16**	***	Species-specific thermal response
**Species model**											
A. coriophora	0.10	8.104	-40.930	3.389	-15.422	3.500	**<2e-16**	***	**1.1e-05**	***	Strong concave chilling response
A. laxiflora	0.02	2.961	2.549	1.136	-4.766	1.080	**0.0249**	*	**1.1e-05**	***	Weak optimum-type response
H. robertianum	0.02	2.801	-5.013	1.212	5.103	1.237	**3.7e-05**	***	**3.9e-05**	***	Strong convex chilling response
H. hircinum	0.05	4.584	-0.001	0.000	NA	NA	**8.5e-06**	***			Linear chilling effect
**(B) Effect of cumulative hours below 12 °C on pre-dormancy tuber mass per vessel**											
**Global model**											
C_{12}^2									**2.4e-07**	***	Strong nonlinear chilling effect
Species									**<2e-16**	***	Strong species differences
C_{12}^2:Species									**1.9e-09**	***	Species-specific thermal response
**Species model**											

A. coriophora	0.18	0.642	-0.000	0.000	NA	NA	**<2e-16**	***			Strong linear chilling response
A. laxiflora	0.09	0.453	0.167	0.250	-1.306	0.253	0.5042		**5.0e-07**	***	Weak optimum-type response
H. robertianum	0.03	0.405	-0.487	0.393	1.104	0.400	0.2165		**0.0062**	**	Convex chilling response
H. hircinum	0.03	0.166	-0.000	0.000	NA	NA	0.0985				No clear chilling effect

Table S7. Linear regression models of the effect of cumulative hours below 10 °C (C_{10}) on post-dormancy tuber mass in g (A) and of cumulative hours below 6 °C (C_6) on post dormancy shoot length in mm (B) of *Anacamptis coriophora, A. laxiflora, Himantoglossum robertianum* and *H. hircinum*. Sample size in *H. hircinum* was insufficient to build a regression model (sample size in Table S2).

**Species**	**Adj-R^2**	**Intercept**	**Lin term**	**Lin SE**	**Lin *p*-value**		**Effect**
**(A) Effect of cumulative hours below 10 °C on post-dormancy tuber mass**							
**Global model**							
C_{10}					**3.9e-07**	***	Strong chilling effect
Species					**<2e-16**	***	Strong species differences
C_{10}:Species					0.3202		No species-specific sensitivity
**Species model**							
A. coriophora	0.02	0.394	-3.7e-05	1.0e-05	**0.0004**	***	Strong negative chilling effect
A. laxiflora	0.05	0.334	-5.2e-05	1.4e-05	**0.0002**	***	Negative chilling effect
H. robertianum	0.45	0.422	-1.3e-04	3.9e-05	**0.0098**	**	Negative chilling effect
H. hircinum	-0.38	0.389	7.5e-05	1.8e-04	0.7187		No chilling effect
**(B) Effect of cumulative hours below 6 °C on post-dormancy shoot length**							
**Global model**							
C_6					**<2e-16**	***	Strong chilling effect
Species					**<2e-16**	***	Strong species differences
C_6:Species					**0.0025**	**	Species-specific sensitivity

**Species model**							
A. coriophora	0.10	3.987	0.003	0.000	**<2e-16**	***	Strong positive chilling response
A. laxiflora	0.12	2.730	0.003	0.000	**1.4e-08**	***	Positive chilling response
H. robertianum	0.81	-0.124	0.009	0.001	**4.1e-05**	***	Very strong chilling response
H. hircinum	0.28	-0.387	0.006	0.004	0.2806		No detectable effect

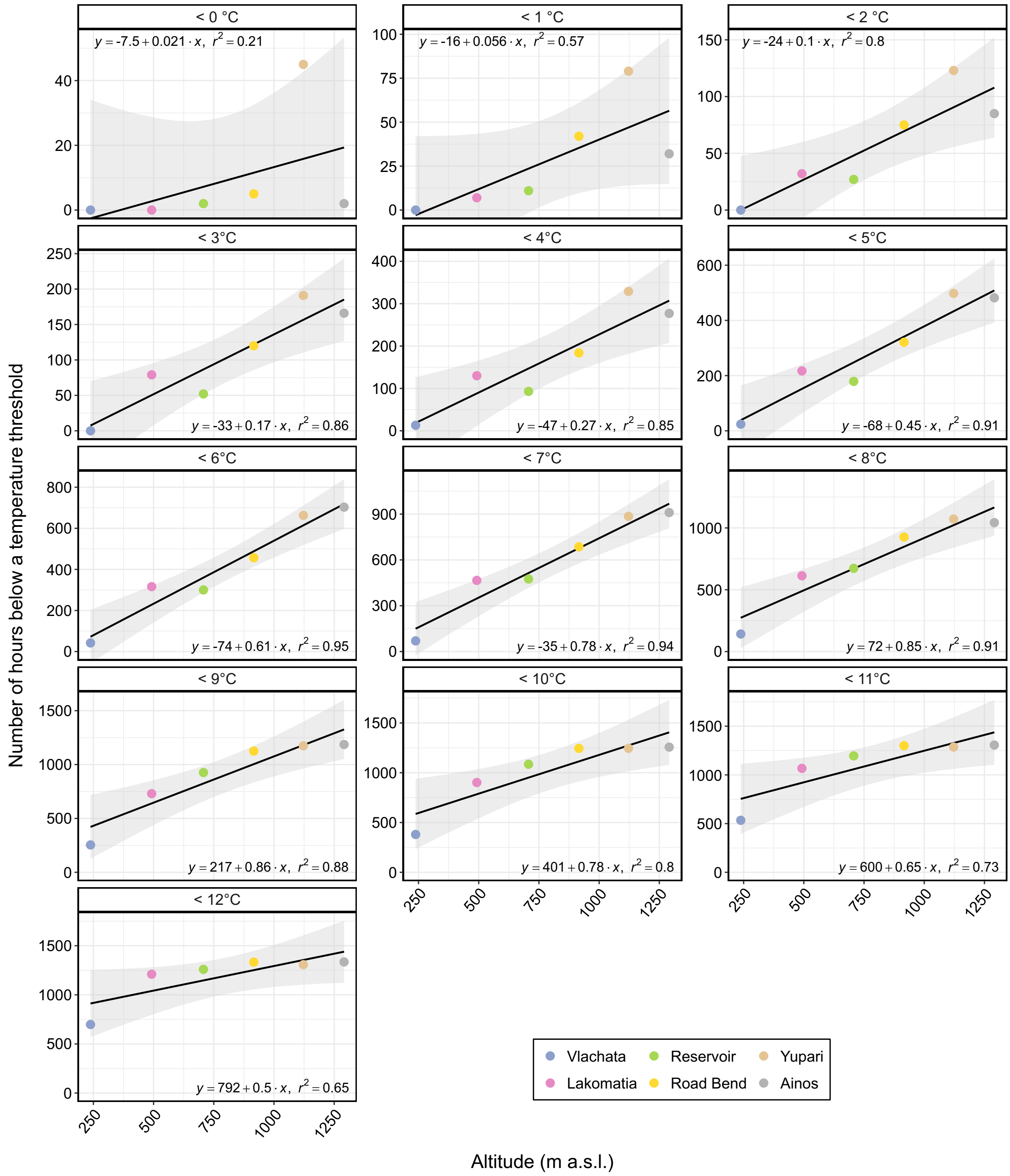

Figure S1. Cumulative hours below a temperature threshold at the end of the cold treatment as a function of altitude above sea level. Each colored point corresponds to an outdoor station. Lines are fitted models.
